# Effect of Carbohydrate Loading in Diabetic Patients Undergoing Laparoscopic Cholecystectomy: A Randomized Controlled Trial

**DOI:** 10.7759/cureus.44570

**Published:** 2023-09-02

**Authors:** Kavita Yadav, Ravi Prakash, Gyan Prakash Singh, Shefali Gautam, Zia Arshad, Brijesh P Singh

**Affiliations:** 1 Anesthesiology, King George's Medical University, Lucknow, IND

**Keywords:** carbohydrate, laparoscopic cholecystectomy, vas, insulin, cortisol, homa-ir, diabetes mellitus, carbohydrate loading

## Abstract

Introduction: Investigations of preoperative oral carbohydrate (CHO) loading have primarily examined benefits among patients without diabetes. Preoperative CHO-rich beverages in general populations have resulted in reductions in insulin resistance after surgery, protein loss, metabolic derangements, and immune dysfunction. The aim of this study was to assess the effect of CHO loading in diabetic patients undergoing laparoscopic cholecystectomy.

Methods: Diabetic patients controlled on oral hypoglycemic agent were randomly divided into two groups: (1) Group T - this group will be given 50 g of maltodextrin before two hours of surgery; (2) Group C - this group will be kept nil per oral as per standard protocol. Blood sugar, serum insulin, serum cortisol, and insulin requirement were compared in both groups.

Results: Blood sugar levels of Group C were found to be significantly higher than that of Group T at six hours and 24 hours. In Group T, a rise in baseline serum insulin (8.94 ± 3.43 mIU/l) was observed at 24 hours (13.23 ± 5.71 mIU/l). A change of 4.29 ± 3.00 mIU/l in serum insulin level was observed. The change in baseline serum insulin levels was 47.99%. In Group C too, a rise in baseline serum insulin (6.27 ± 1.74 mIU/l) was observed at 24 hours (18.00 ± 5.34 mIU/l). A change of 11.73 ± 4.97 mIU/l in serum insulin level was observed. The change in baseline HOMA-IR (homeostatic model assessment for insulin resistance) levels in Group T was 53.66%. A rise (4.39 ± 1.63) in baseline HOMA-IR of Group C (1.65 ± 0.45) was observed at 24 hours (6.04 ± 1.76). The change in baseline HOMA-IR levels in Group C was 266.06%.

Conclusions: CHO loading is observed to be beneficial in diabetic patients undergoing laparoscopic cholecystectomy. No adverse effects or an increased risk of aspiration were observed in the intervention group during the study period.

## Introduction

For several decades, surgery has been conducted with preoperative fasting allowing for safe anesthesia induction with minimal risk of aspiration. However, the literature has suggested that preoperative fasting exaggerates the physiological stress of the surgery, both of which are sufficient to cause increased catabolism in the body, depleting the glucose reserves and enhancing insulin resistance [1]. Investigations of preoperative oral carbohydrate (CHO) loading have primarily examined benefits among patients without diabetes. Preoperative CHO-rich beverages in the general population have resulted in reductions in insulin resistance after surgery, protein loss, metabolic derangements, and immune dysfunction [2,3]. In fact, insulin resistance has been reduced by 50% among patients without diabetes who consume preoperative CHO beverages. There has been a reduction in the chances of hyperglycemia along with the maintenance of lean body mass, muscle strength, and neutral nitrogen balance [4-6]. Researchers of the preoperative CHO loading also observed an increase in patient satisfaction [7,8]. The present randomized study was conducted to evaluate the effects of CHO loading in patients undergoing elective surgery (laparoscopic cholecystectomy).

## Materials and methods

Study design

This was an open-label randomized control trial.

Sample size

It was calculated on the basis of a reduced risk ratio of no effects on in-hospital complications (nausea/vomiting) using the formula: [z2 * p(1-p)] / e2 / 1 + [z2 * p(1-p)] / e2 * N]; where p1 = 0.88, the reduced risk ratio in study group; p2 = 1.0, under null hypothesis; risk ratio e = 0.15, the risk ratio reduction considered to be clinically significant. Type I error α = 5% (level of significance). Type II error β = 20% for setting power of study 80%. The sample size was calculated to be n = 32.

Study setting

After approval from the institutional ethical committee (human) (102 ECM II B-THESIS/P79) and Clinical Trials Registry-India (CTRI) registration (2021/01/030718), this study was conducted in a tertiary care teaching hospital over a one-year duration.

Method of randomization

Randomization was done using a computer-generated random number table.

Participants

Diabetic patients aged 18-55 years, with American Society of Anesthesiologists physical status II, BMI between 18 and 35 kg/m2, who were adequately controlled on oral hypoglycemic drugs (glycosylated hemoglobin, fasting, and post-prandial blood sugar within normal limits), and were undergoing elective laparoscopic cholecystectomy were included in this study. Patients with any complication of diabetes or requiring insulin, pregnant patients, patients with gastroesophageal reflux disease, hiatus hernia, or any other condition with increasing risk of aspiration, patients on steroids and chronic opioid use, or any major complication occurring during surgery or surgery exceeding four hours were excluded from the study.

Intervention: study groups

The study was conducted among two groups: (1) Group T - this group was given 50 g of maltodextrin (CHO) in 400 ml of water two hours before surgery; (2) Group C - this group was kept nil per oral (NPO), as per standard practice.

In this study, both groups were asked to be NPO for at least eight hours before surgery, as per standard guidelines. Group T was asked to drink maltodextrin 50 gm dissolved in 400 ml water two hours before induction of anesthesia. Group C was just kept NPO. The blood samples for blood sugar, serum insulin, and serum cortisol were taken before giving maltodextrin. The case under study was the first case to be taken up in the operation theater (OT).

In the OT, the patient was induced by giving general anesthesia as per standard protocols, i.e., premedication by giving intravenous midazolam (1 mg) and fentanyl (2 mcg/kg); induction by giving intravenous propofol (1.5-2.5 mg/kg) and vecuronium (0.08-0.1 mg/kg); and maintenance by inhalational gases, i.e., oxygen, nitrous oxide, sevoflurane, and intermittent doses of intravenous vecuronium (0.01-0.02 mg/kg). An intraoperative balanced isotonic solution, i.e., Sterofundin^TM^ was used. Intraoperatively, Ryle’s tube was aspirated 10 minutes after induction of anesthesia, and the volume and pH (using an electronic pH meter) of the gastric contents were measured. Reversal of anesthesia was done with intravenous neostigmine (0.04-0.08 mg/kg) and glycopyrrolate (0.005-0.01 mg/kg).

Outcome

A total of four blood sugar readings were taken: (1) before giving maltodextrin; (2) before induction of anesthesia (i.e., two hours after giving oral maltodextrin); (3) six hours after giving maltodextrin; and (4) 24 hours after maltodextrin on the first postoperative day (fasting).

Regular insulin was given only if blood sugar was >180 (according to the sliding scale). On the morning of the first postoperative day, serum insulin and serum cortisol samples were sent along with blood sugar samples.

Statistics

Data collected were analyzed using SPSS software version 27 (IBM Corp., Armonk, NY). Student's T-test was used to compare paired parametric data. The chi-square test was used to compare categorical data. P-value <0.5 was considered significant.​​​​​​​

## Results

**Figure 1:**
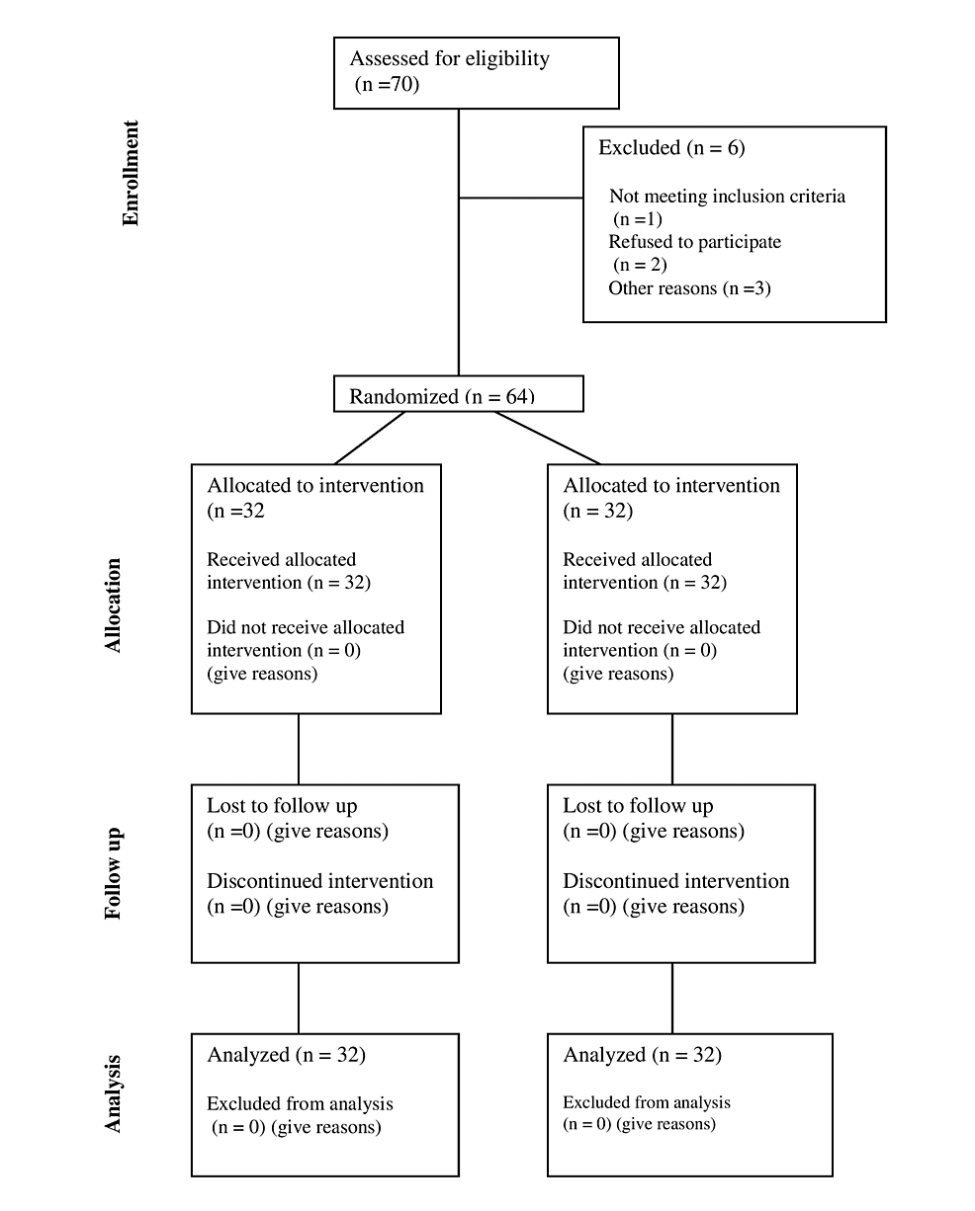
Consolidated Standards of Reporting Trials (CONSORT) flow diagram

Out of 64 patients enrolled in the study, 32 (50.0%) were subjected to CHO loading (50 g maltodextrin dissolved in 400 ml of water before two hours of surgery) and were classified as the test group (Group T), and the rest 32 (50.0%) were kept NPO and were classified as the control group (Group C) (Figure 1). The mean age of patients enrolled in the study ranged was 43.08 ± 8.60 years. The mean age of patients in Group T (intervention) was 42.53 ± 8.88 years while that in Group C (control) was 43.63 ± 8.41 years. Anthropometric values were comparable in both groups (Table 1).

**Table 1:** Comparison of baseline anthropometric parameters

S. No.	Anthropometric characteristics	Group T (n = 32)		Group C (n = 32)		Student's t-test	
		Mean	SD	Mean	SD	t	p
1	Weight (kg)	61.34	7.32	62.28	7.05	-0.522	0.604
2	Height (cm)	159.44	6.29	160.22	7.53	-0.450	0.654
3	BMI (kg/m^2^)	24.23	2.21	24.22	1.73	0.008	0.993

Blood sugar levels in both groups are shown in Table 2. Significant differences were present in two, six, and 24 hours. In both groups, a statistically significant decline in baseline serum cortisol level was observed after 24 hours. The percentage change in baseline serum cortisol levels of Group T was 23.43% (4.59 ± 1.10 mcg/dl) while that in Group C was 27.77% (4.41 ± 0.71 mcg/dl) (Table 3).

**Table 2:** Comparison of blood glucose levels at different time intervals

S. No.	Time interval	Group T (n = 32)		Group C (n = 32)		Student's t-test	
		Mean	SD	Mean	SD	t	p
1	Baseline: Before maltodextrin administration (fasting)	109.59	10.45	106.38	7.73	1.401	0.166
2	2 hours after baseline (before induction)	136.03	9.01	102.50	6.84	16.772	<0.001
3	6 hours after baseline	135.25	7.48	150.28	12.88	-5.710	<0.001
4	24 hours after baseline (fasting)	112.91	12.30	136.22	5.07	-9.917	<0.001
	Intragroup change in baseline random blood sugar ± SD (% change) after 24 hours	3.31 ± 7.30 (3.02%)		29.84 ± 8.90 (28.05%)			
	Paired t-test	t = 2.568; p = 0.015		t = 18.974; p < 0.001			

**Table 3:** Intragroup change in baseline serum cortisol at 24 hours

S. No.	Time period	Group T (n = 32)		Group C (n = 32)	
		Mean	SD	Mean	SD
1	Baseline	19.63	3.08	15.88	2.99
2	24 hours	15.03	2.78	11.47	3.04
	Mean change ± SD in baseline serum cortisol (% change)	-4.59 ± 1.10 (23.43%)		-4.41 ± 0.71 (27.77%)	
	Paired t-test	t = -23.558; p < 0.001		t = -35.004; p < 0.001	

In Group T, a rise in baseline serum insulin (8.94 ± 3.43 mIU/l) was observed at 24 hours (13.23 ± 5.71 mIU/l). A statistically significant change of 4.29 ± 3.00 mIU/l in serum insulin level was observed. The percentage change in baseline serum insulin levels was 47.99%. In Group C too, a rise in baseline serum insulin (6.27 ± 1.74 mIU/l) was observed at 24 hours (18.00 ± 5.34 mIU/l). A statistically significant change of 11.73 ± 4.97 mIU/l in serum insulin level was observed. The percentage change in baseline serum insulin levels was 187.08%. The percentage change in baseline insulin level in Group T was only 47.99% and the same in Group C was 187.08% (Table 4).

**Table 4:** Comparison of intraoperative gastric volume and pH

S. No.	Intraoperative gastric characteristics	Group T (n = 32)	Group C (n = 32)
1	Mean volume ± SD (range)	13.97 ± 4.86 (5.0-20.0)	11.05 ± 3.97 (5.0-19.0)
	Student's t-test	t = 2.635; p = 0.011	
2	Mean pH ± SD (range)	6.63 ± 0.27 (5.9-6.9)	4.02 ± 0.34 (3.5-4.8)
	Student's t-test	t = 34.057; p < 0.001	

A statistically significant rise (1.32 ± 0.96) in baseline HOMA-IR (homeostatic model assessment for insulin resistance) of Group T (2.46 ± 1.09) was observed at 24 hours (3.77 ± 1.89). The percentage change in baseline HOMA-IR levels in Group T was 53.66%. A statistically significant rise (4.39 ± 1.63) in baseline HOMA-IR of Group T (1.65 ± 0.45) was observed at 24 hours (6.04 ± 1.76). Percentage change in baseline HOMA-IR levels in Group C was 266.06% (Table 5).

**Table 5:** Intragroup change in baseline HOMA-IR (homeostatic model assessment for insulin resistance) levels at 24 hours

S. No.	Time period	Group T (n = 32)		Group C (n = 32)	
		Mean	SD	Mean	SD
1	Baseline	2.46	1.09	1.65	0.45
2	24 hours	3.77	1.89	6.04	1.76
	Mean change ± SD in baseline serum insulin (% change)	1.32 ± 0.96 (53.66%)		4.39 ± 1.63 (266.06%)	
	Paired t-test	t = 7.729; p < 0.001		t = 15.228; p < 0.001	

Group T as compared to Group C had significantly higher intraoperative gastric volume (13.97 ± 4.86 vs. 11.05 ± 3.97 cc) and intraoperative gastric pH (6.63 ± 0.27 Vs. 4.02 ± 0.34) (Table 6).

**Table 6:** Comparison of intraoperative gastric volume and pH

S. No.	Intraoperative gastric characteristics	Group T (n = 32)	Group C (n = 32)
1	Mean volume ± SD (range)	13.97 ± 4.86 (5.0-20.0)	11.05 ± 3.97 (5.0-19.0)
	Student's t-test	t = 2.635; p = 0.011	
2	Mean pH ± SD (range)	6.63 ± 0.27 (5.9-6.9)	4.02 ± 0.34 (3.5-4.8)
	Student's t-test	t = 34.057; p < 0.001	

There were no reports of dizziness. The incidence of vomiting was similar in both groups (12.5%). Incidence of nausea was higher in Group C as compared to Group T (31.3% vs. 21.9%) but this difference was not found to be significant statistically. The range of comfort VAS (visual analog scale) in Group T was 1-4 (median 2), while that in Group C was 2 to 4 (median 3). The mean comfort VAS of Group T (1.97 ± 0.82) was found to be significantly lower than that of Group C (2.81 ± 0.82).

## Discussion

The essential anabolic hormone of the body is insulin. Once the stress of post-surgery starts, the body becomes insulin resistant (IR), and the catabolic state starts. In the catabolic state, protein, CHO, and fats are utilized to assist in the healing and synthesis of tissue and other substances like acute-phase proteins. There are two areas that are most impacted by insulin resistance: liver and peripheral structures, typically muscles. Peripheral IR decreases the uptake of glucose, which causes hyperglycemia, whereas hepatic IR causes gluconeogenesis [5].

In our study, serum insulin levels on the first postoperative day in the control group were found to be significantly higher than that of the intervention group (18.00 ± 5.34 vs. 13.23 ± 5.71 mIU/l). Similarly, the HOMA-IR of the control group was found to be significantly higher than that of the intervention group (4.39 ± 1.63 vs. 3.77 ± 1.89). Therefore, suggesting a higher insulin sensitivity (IS) in the intervention group in comparison to the control group. Nygren et al. conducted a study on 30 patients undergoing either colorectal resection (n = 16) or total hip replacement (THR) (n = 14) where the administration of preoperative CHO loading was assessed against the placebo group [9]. Insulin resistance was assessed, and it was found that in patients of the THR group, there was a 37% reduction in IS in the placebo group immediately after surgery. No significant reduction in IS was found in the CHO group. In the colorectal surgery group, there was a 24% greater reduction in IS in the fasted group than in the CHO group at 24 hours after surgery.

Svanfeldt et al. conducted a study where insulin resistance was assessed, and it was found that IS was significantly increased by 50% three hours after morning drink (p < 0.01) [7]. Faria et al. conducted a study on 21 patients undergoing laparoscopic cholecystectomy where it was found that IS was significantly higher in the intervention group than the control group [10]. Okabayashi et al. conducted a study on 26 patients undergoing hepatic resection where it was found that IS was significantly better in the intervention group than the control group [11]. Rizvanović et al. conducted a study on 50 patients undergoing colorectal resection where it was found that IS was significantly higher in the intervention group than the control group [12]. The study also assessed the well-being by VAS score and found it to be comparable in both groups. The findings of our study are comparable with the various studies conducted on non-diabetic patients undergoing laparoscopic cholecystectomy as well as other surgeries. The findings are suggestive of the benefits of preoperative CHO loading.

The degree of the stress response can be calculated through many ways like measuring the hormones (growth hormone, insulin, catecholamines, plasma concentration of cortisol, etc.), neuroendocrine sequelae, and other metabolic changes (nitrogen loss and hyperglycemia). In our study, serum cortisol levels showed a statistically significant decline in baseline serum cortisol levels after 24 hours in the intervention group. Zelic et al. conducted a study including patients who underwent laparoscopic cholecystectomy where there was a significant increase in the postoperative serum C-reactive protein (CRP) in both groups; however, the increase was more in the group with fasting protocol both 24 and 48 hours postoperative [13]. In fed patients, cortisol concentration measured in the afternoon immediately after the operation showed a physiological decline. In patients with fasting protocol, postoperative cortisol values rise above the values measured in the morning. Preoperative feeding is more advantageous than overnight fasting by decreasing preoperative discomfort among patients following laparoscopic cholecystectomy.

Pedziwiatr et al. conducted a study on 40 patients undergoing laparoscopic cholecystectomy where it was found that the levels of cortisol, insulin, and insulin resistance were different, but not statistically significant [14]. The length of stay (LOS) and postoperative complications were similar. Similar to our findings, serum cortisol levels were reduced in the intervention group in the study conducted by Zelic et al. on non-diabetic patients undergoing laparoscopic cholecystectomy [13].

In patients without diabetes, CHO-loading beverages improved perioperative glycemic management with a lower chance of hyperglycemia, preserved lean body mass, maintained muscle strength, and neutral nitrogen balance. In our study, the blood glucose levels of the control group were found to be significantly higher than that of the intervention group at different periods of observation of blood glucose, i.e., two hours, six hours, and 24 hours, respectively. Although, at 24 hours, the mean blood sugar level was found to be above baseline levels in both the groups, the control group had a significantly higher value than the intervention group on the first postoperative day, suggesting the benefits of preoperative CHO loading. Weledji et al. conducted a hospital-based prospective case-control study on 70 patients undergoing elective surgery where the administration of preoperative CHO loading was assessed against a control group. It was found that the control group had significantly higher mean blood glucose on the first postoperative day (146.20 ± 38.36 mg/dl vs.123.06 ± 26.64 mg/dl, p = 0.004) and postoperative infections (31.43% vs. 8.57%) and longer mean length of hospital stay (12.54 days vs. 9.17 days) although the difference was not statistically significant [15]. Results showed that preoperative CHO loading is effective and safe in reducing postoperative hyperglycemia and infection in open general surgery even in a low-resource setting. The results of our study are comparable with the above study conducted on other elective surgeries.

The safety of 400 ml of CHO loading is a crucial factor for anesthetists. Maltodextrin (complex CHO and a constituent of the CHO drink) takes more time in comparison to water in gastric emptying. Still, it empties from the stomach only within 90 minutes (as per gamma camera assessment). As per recent studies, either co-administration of paracetamol (with successive measurement of serum paracetamol concentrations) or ultrasound assessment is used for gastric emptying. Overall, it was noticed that CHO loading is a safe method with no incidence of pulmonary aspiration in a large meta-analysis and study of five million patients globally who were given CHO as part of the ER program [16].

In our study, the intervention group as compared to the control group had higher intraoperative gastric volume (13.97 ± 4.86 vs. 11.05 ± 3.97 cc) and intraoperative gastric pH (6.63 ± 0.27 vs. 4.02 ± 0.34). This amount of volume is considerably safe for patients and no hazardous risks of aspiration were observed during this study. The gastric pH was also less acidic in the intervention group. Nygren et al. conducted a study on 12 patients undergoing laparoscopic cholecystectomy and parathyroid surgery, where the administration of preoperative CHO loading was assessed [7]. The study assessed gastric emptying by the technique of gamma cameras and a radiotracer mixed with the drink and found that there was no significant difference in gastric fluid volume (GFV). The duration of GFV in the CHO group was 90 minutes. The study also assessed the well-being of the patient by VAS, and it was found that thirst was decreased during the first 60 minutes after CHO and 40 minutes after water. Thereafter, no significant changes were observed. Hunger was reduced after 20 minutes of water but not after CHO. Anxiety was reduced after water but not after CHO.

Yagci et al. conducted a study on 70 patients undergoing laparoscopic cholecystectomy and thyroidectomy, where the study assessed gastric emptying by the technique of nasogastric tube and found that there was no significant difference in GFV. Gastric acidity was also assessed through the technique of a urine pH meter, and it was found that it was comparable in all the study groups [15]. The beneficial effects of our study are comparable with the above study.

Enhanced recovery after surgery (ERAS) pathways incorporate preoperative CHO loading, which reduces postoperative nausea and emesis and enhances the return of gut function [6]. In our study, none of the patients complained of dizziness. The incidence of vomiting was similar in both groups. The incidence of nausea was higher in the control group as compared to the intervention group, but this difference was not found to be significant statistically. The mean comfort VAS of the intervention group was found to be significantly lower than that of the control group. Hausel et al. conducted a study on 172 patients undergoing laparoscopic cholecystectomy where the study assessed the well-being of the patient by VAS and objective analysis of nausea and vomiting by nursing staff, and it was found that the incidence of nausea and vomiting was comparable in the three groups during the first 12 hours [16]. Between 12 and 24 hours, more patients in the fasted group experienced nausea and vomiting than in the CHO group. Our study had a comparable incidence of postoperative nausea and vomiting but the mean VAS was lower in the intervention group.

Diabetes mellitus (DM) is a major health issue globally affecting both developing and developed countries. The presence of unnoticed and uncontrolled hyperglycemia in DM may result in a variety of complications over time. A major concern arises when diabetic patients are undergoing elective or emergency surgeries or hospitalization for diverse etiologies related or unrelated to DM. It has been seen that diabetic patients have an elevated risk of adverse surgical outcomes, high morbidity, and high mortality rates. Many elective surgical patients have diabetes and such patients demand complex diabetes care and management for a better outcome.

It was a single-center study, and we would need more supporting data on diabetic patients to follow it as a part of the ERAS protocol. The study is limited to short-duration surgeries and laparoscopic surgeries, so it would be difficult to compare data with long-duration surgeries and open/major surgeries.

## Conclusions

CHO loading is observed to be beneficial in diabetic patients undergoing laparoscopic cholecystectomy. Preoperative CHO improves insulin resistance and provides a sense of well-being to the patient. No major adverse effect is seen with preoperative CHO ingestion. There is no increase in the risk of aspiration. We recommend routine use of preoperative oral CHO in well-controlled diabetic patients.

## References

[1] Awad S, Varadhan KK, Ljungqvist O, Lobo DN (2013). A meta-analysis of randomised controlled trials on preoperative oral carbohydrate treatment in elective surgery. Clin Nutr.

[2] Barclay KL, Zhu YY, Tacey MA (2015). Nausea, vomiting and return of bowel function after colorectal surgery. ANZ J Surg.

[3] Makuuchi R, Sugisawa N, Kaji S (2017). Enhanced recovery after surgery for gastric cancer and an assessment of preoperative carbohydrate loading. Eur J Surg Oncol.

[4] Talutis SD, Lee SY, Cheng D, Rosenkranz P, Alexanian SM, McAneny D (2020). The impact of preoperative carbohydrate loading on patients with type II diabetes in an enhanced recovery after surgery protocol. Am J Surg.

[5] Nygren J, Thorell A, Jacobsson H, Larsson S, Schnell PO, Hylén L, Ljungqvist O (1995). Preoperative gastric emptying. Effects of anxiety and oral carbohydrate administration. Ann Surg.

[6] Fawcett WJ, Ljungqvist O (2017). Starvation, carbohydrate loading, and outcome after major surgery. BJA Education.

[7] Svanfeldt M, Thorell A, Hausel J, Soop M, Nygren J, Ljungqvist O (2005). Effect of "preoperative" oral carbohydrate treatment on insulin action--a randomised cross-over unblinded study in healthy subjects. Clin Nutr.

[8] Gustafsson UO, Nygren J, Thorell A, Soop M, Hellström PM, Ljungqvist O, Hagström-Toft E (2008). Pre-operative carbohydrate loading may be used in type 2 diabetes patients. Acta Anaesthesiol Scand.

[9] Nygren J, Soop M, Thorell A, Sree Nair K, Ljungqvist O (1999). Preoperative oral carbohydrates and postoperative insulin resistance. Clin Nutr.

[10] Faria MS, de Aguilar-Nascimento JE, Pimenta OS, Alvarenga LC Jr, Dock-Nascimento DB, Slhessarenko N (2009). Preoperative fasting of 2 hours minimizes insulin resistance and organic response to trauma after video-cholecystectomy: a randomized, controlled, clinical trial. World J Surg.

[11] Okabayashi T, Nishimori I, Yamashita K (2010). Preoperative oral supplementation with carbohydrate and branched-chain amino acid-enriched nutrient improves insulin resistance in patients undergoing a hepatectomy: a randomized clinical trial using an artificial pancreas. Amino Acids.

[12] Rizvanović N, Nesek Adam V, Čaušević S, Dervišević S, Delibegović S (2019). A randomised controlled study of preoperative oral carbohydrate loading versus fasting in patients undergoing colorectal surgery. Int J Colorectal Dis.

[13] Zelić M, Štimac D, Mendrila D, Tokmadžić VS, Fišić E, Uravić M, Šustić A (2013). Preoperative oral feeding reduces stress response after laparoscopic cholecystectomy. Hepatogastroenterology.

[14] Pędziwiatr M, Pisarska M, Matłok M (2015). Randomized clinical trial to compare the effects of preoperative oral carbohydrate loading versus placebo on insulin resistance and cortisol level after laparoscopic cholecystectomy. Pol Przegl Chir.

[15] Weledji EP, Njong SN, Chichom A, Verla V, Assob JC, Ngowe MN (2017). The effects of preoperative carbohydrate loading on the metabolic response to surgery in a low resource setting. Int J Surg Open.

[16] Yagci G, Can MF, Ozturk E, Dag B, Ozgurtas T, Cosar A, Tufan T (2008). Effects of preoperative carbohydrate loading on glucose metabolism and gastric contents in patients undergoing moderate surgery: a randomized, controlled trial. Nutrition.

